# A Holistic, Person-Centred Care Model for Victims of Sexual Violence in Democratic Republic of Congo: The Panzi Hospital One-Stop Centre Model of Care

**DOI:** 10.1371/journal.pmed.1002156

**Published:** 2016-10-11

**Authors:** Denis Mukwege, Marie Berg

**Affiliations:** 1 Panzi General Referral Hospital, Bukavu, Democratic Republic of Congo; 2 Panzi Foundation, Bukavu, Democratic Republic of Congo; 3 Institute of Health and Care Sciences, Sahlgrenska Academy, University of Gothenburg, Gothenburg, Sweden; 4 Centre for Person-Centred Care (GPCC), University of Gothenburg, Gothenburg, Sweden

## Abstract

Denis Mukwege and Marie Berg describe the One Stop Centre at Panzi Hospital in Eastern Democratic Republic of Congo that provides care for girls and women who have been raped in combination with extreme bodily harm.

**Summary Points**

- One-Stop Centre (OSC) is an innovative, holistic, person-centred care model developed in recent years for survivors of violence against women and girls.
- OSC at Panzi Hospital in eastern Democratic Republic of Congo has been developed after years of treating girls and women who have been raped in combination with extreme bodily harm.
- OSC comprises four pillars, covering medical, psychosocial, legal, and socioeconomic care needs, which are fulfilled in partnership. Based on genuine listening to a harmed girl’s or woman’s personal narrative, personalised care is planned, implemented, and documented with the aim of achieving health and reintegration in society.
- OSC gives more than holistic individual care; it provides a platform for achieving a healthy life at the micro- (the person) and meso- (local society) levels and, if conscientiously and systematically implemented in all health care structures, facilitates achievement of the right to health for all on the macro (national) level.

## The Challenge

The provision of sexual and reproductive rights and health is an important component in ensuring the highest attainable standard of health [1,2]. However, this is a challenge in the Democratic Republic of Congo (DRC), especially in the eastern part, where rape of women and girls, with extreme sexual violence, has been a leading cause of individual and societal suffering in the last decades. Rape in combination with extreme bodily harm has been used as a war tactic by armed groups and has escalated as a new pathologic societal behaviour among civilians. The sexual and bodily violence related to rapes is not only about destruction of women’s physical and mental functions; it is about the right to health and socioeconomic life of a society [3,4].

The Panzi General Referral Hospital in the Ibanda Health Zone in Bukavu, South Kivu, eastern DRC, has, from its start in 1999 through to 2015, treated 48,482 women following extreme sexual violence. Since 2004, this care is organised under the Survival of Sexual Violence (SSV) project. In addition, 37,382 women received treatment for gynaecological problems after complicated childbirth or poor medical treatment, of which many were the results of rape [5]. This experience has developed the medical skills of staff, enabling them to treat severe conditions, such as vesicovaginal fistulas, that they would rarely have seen elsewhere. It has also identified that medical care alone is not enough to heal, comfort, and restore the needs of suffering girls and women [3]. For holistic recovery and achieving a healthy life, a wider holistic care model is needed. In this article, we describe how a “One-Stop Centre (OSC) Care” model has developed to provide holistic care and complementary services in Bukavu and surrounding health care facilities and communities. Our backgrounds are as follows: DM is director of the Panzi Hospital, a gynaecologist, and an obstetrician who has, for 15 years, treated numerous patients with sequelae after extreme sexual violence and complicated childbirth or complications after childbirths managed at low-quality maternity care settings; and MB is a professor in Health Care Sciences specialising in reproductive and perinatal health and a registered nurse midwife with a long experience of working in DRC, but not specifically with the survivors of sexual violence.

## The One-Stop Centre Care Model

OSC models of care have, in recent years, been developed globally in several settings for survivors of violence against women and girls, especially as a method for scaling up quality services during post-conflict reconstruction and recovery in low-income countries [6]. Methodologies are being refined to extend and improve services for survivors as well as to build the capacity of local organisations to take on the issue [7].

Successively, to the extent possible given available resources, the OSC care philosophy is being established at the Panzi Hospital and in services run by the connected Panzi Foundation. The OSC care model is developed to be a holistic, person-centred system of care aimed at covering a woman’s essential needs for recovery and (re)gaining of a healthy life, in particular those women harmed by extreme sexual violence or a complicated childbirth. It offers a variety of competences and activities in Bukavu and other health care facilities in a vast geographic area.

The Panzi OSC care model follows generic standards and consensus for what has been defined as good health care [8]. It is structured in four pillars: medical, psychosocial, legal, and socioeconomic care. Its basic standpoint is that empowerment of women is the foundation for constructing a plausible and prosperous society. It has much in common with a Person-Centred Care (PCC) approach, based on what is and what constitutes a human [9] and a person [10,11]: a kind of lifeworld-led health care [12], with essentialities common with the PCC model for persons with long-lasting conditions and chronic diseases [13]. It treats women who seek care as a dignified person (not an object) with value, rights, will, and capabilities, which necessitates trying to understand the situation as the woman understands it. This is done first by carers carefully and actively listening to her narrative and identifying her care needs according to the four pillars, in contrast to the extreme, undignified treatment the harmed women have experienced previously in life. Second, the care plan in any of the pillars is developed in partnership with and decided in agreement between the woman and the professional specialist (psychologists, lawyers, economists, and other professionals needed for holistic care). This is shown in activities such as informing, explaining, and providing support in decision-making, such as whether to have gynaecological surgery or to process juridical action against perpetrators of sexual violence. Third, the partnership is safeguarded through documentation of all care in structured care plan templates. Several protocols for the different pillars of care are developed and used to ensure that all needs are explored and performed as necessary and/or wanted.

The person-centred OSC model of care requires a multi-professional team comprising resources such as doctors, nurses, midwives, laboratory technicians, radiology technicians, pharmacy assistants, lawyers, paralegals, administrative resources, and people facilitating women’s reintegration in society. As applied at Panzi Hospital, with its high capabilities in human resources, equipment, and infrastructures, it cannot be exactly replicated in resource-limited areas. A reduced and adapted OSC model, which brings it closer to communities, has been designed and is functioning in some rural areas (such as in Mulamba and Bulenga).

The care model described in this paper focuses on the majority of patients, who are adult women. As in other settings, sexual violence towards males is also a reality in DRC; 1.5% of the patients within the Panzi OSC are men. In addition, specific care is given for minors by staff trained in treating children such as paediatricians, paediatric surgeons, child psychiatrists, and psychologists. Specially adapted child examination rooms with appropriate equipment have been developed. In specific cases, such as minors who experience rape by family members and, after receiving care, are exposed again to the same individuals and the same incidents, the SSV child is separated from this family member and is hosted together with her mother or family carer in a transit centre while legal proceedings are initiated. The OSC also organises sessions of family mediation and reunification. On a community level, its paralegals organise awareness sessions and education on human rights and collective protection.

### Access to the One-Stop Centre

All patients in need of treatment and support for regaining sexual and reproductive health are welcome to the Panzi OSC. They arrive through a variety of channels, although the majority enter through the health care system. Many are referred by international and local partner associations of the SSV project in territories of the South Kivu province. Others are identified by the Panzi Foundation’s mobile clinic during its field missions by its specifically trained para-juridical assistants or by the police. Some arrive themselves or with support of relatives.

The starting point of the OSC care model is the medical pillar, around which revolves psychosocial services, legal support, and socioeconomic reintegration to ensure holistic care in one place. This spares the patient from repeating her narrative to every service she needs. Upon admission, the patient is registered by a coordinating psychosocial worker. This includes listening to and documenting the woman’s narrative, identifying primary needs, and assigning a personal psychosocial worker, a nurse, who remains her contact person, leading and coordinating the woman’s care plan and treatments throughout her stay. Care is performed both at the hospital as well as in so-called “transit care houses” just outside the hospital, in which patients coming from far away can stay until they are ready to return to their communities. Patients can, after first treatment started at the Panzi Hospital, get further care in an OSC closer to their homes.

### Medical Care

Medical care starts with a physician consultation, including a medical examination and plan of further examinations, such as tests for HIV, pregnancy, syphilis, hepatitis B, and other tests for identifying secondary effects of bodily harm, such as ultrasound and radiography. This effectively determines needed care. Patients are treated on the basis of two different protocols depending on the amount of time that has passed since the sexual violence incident. For patients for whom a long time has passed since the sexual violence incident, medical care becomes more complex and the psychological sequelae become more important. Post-exposure prophylaxis for HIV and sexually transmitted diseases is provided for women arriving within 72 hours, which is about 10% of the patients, and curative care is given for those arriving more than 72 hours after the sexual violence incident.

Recommended care is explained to the patient who, in a shared decision-making process, signs a consent form and the defined health care plan, allowing the doctors to proceed with examination. If needed, the woman is referred to a specialist in gynaecology, cardiology, internal medicine, radiology, or paediatrics.

### Psychosocial Care

Psychosocial care starts with meeting a psychologist to identify needs and plan for specific treatment, which is carried out individually and in groups. This is in addition to various activities offered to all women, including drama and music therapy as well as occupational therapy activities such as basket-making, flower arranging, sewing, and knitting. Such activities promote processing of experiences and provide relief and feelings of value. Women suffering from post-traumatic stress disorders, depression, anxiety, and other psychiatric disorders receive specialist neuropsychiatric treatment. Psychosocial care also, when necessary, includes counselling for a woman’s close relatives. This could be for a husband to overcome anger, bitterness, and blame related to an incident of sexual violence, couple counselling, such as after domestic violence, and counselling of next-of-kin to prevent marginalising the harmed woman.

### Legal Care

Legal care aims to support women in deciding whether or not to take legal action against their attacker. This includes actively listening to her narrative to inform and support consciousness of her human rights, to analyse the situation together, and to support her decision in how to proceed. This strengthens the woman’s courage and capacity to act, including expressing herself officially and starting a legal process against a perpetrator of sexual or domestic violence. It also includes assistance with transport to the place where a perpetration/violence has occurred, arrangement to meet the perpetrator(s), and transport of a perpetrator to tribune in order to carry out a trial. The decision to start a legal process is always decided upon and signed by the woman herself.

### Socioeconomic Care

For most of the women, traumatic experience marginalises them from family and society and destroys their economic capability. The socioeconomic care pillar safeguards a woman’s essential ability for healthy living, with the view that every woman, regardless of condition severity, is a resource for herself and society. The need for additional life skills training is identified, and activities are offered in the transit house (“Dorkas”). These include literacy and mathematics, household maintenance skills, hygiene, and nutrition. They also gain skills to provide economically for their household. In “City of Joy,” women with leadership personalities are also offered women’s activist training.

Socioeconomic actions redevelop the woman’s social network, because sharing similar stories creates strong sisterhood links. Other activities and projects help women, upon return to their home village, with starting “microfinancing collectives” in groups with a maximum of 30 people led by one of the women with leadership capacity. Each member has to contribute something: for example, an amount of money or cultivation on a commune field. The collective members support each other and get a proportion of the gains at year-end according to their contribution.

### Reintegration in Society

Part of treatment is to plan for and enhance the woman’s return to her community. Follow-up home visits after leaving the hospital are organised by teams to assess and secure the reintegration. This includes providing family mediation for those with difficult reintegration, counselling for couples, psychological support, guidance on medication use, and identification of additional care needs in any of the four pillars. It also encourages the woman to be part of organised community collectives, such as microfinancing. In Box 1, a fictionalised case narrative illustrates the OSC care model in practice.

**Box 1. A Fictionalised Case Narrative Illustrating the One-Stop Centre Care Model in Practice**

The individuals and events described below are not real but provide a realistic representation of the type of patients encountered and services that are provided.

- Furaha, a 17-year-old single woman from a town 220 km south of Panzi Hospital, is referred by a nongovernmental organisation. In the hospital reception triage, she is identified as needing treatment as SSV. The SSV social assistant coordinator registers her attendance, completes a short demographic document, and chooses a social assistant to be responsible for coordinating the care of Furaha. In a first meeting, the social assistant listens to Fuhara´s narrative, documents it, and, in agreement with Furaha, makes a written care plan covering her needs, including examination by a gynaecologist and a psychologist.
- *The narrative*: Seventeen months earlier, Furaha was sleeping at her family home when two plain-clothes armed military men suddenly entered the house. They spoke the local language and another language often used by militaries; she presumed they were members of a local rebellion movement. They took Furaha forcibly, undressed her, and sexually raped her several times before leaving. Her mother, father, and older siblings woke up during the incident but did not dare to act. Two months later, Furaha found that she was pregnant. She tried to hide it from her family, but it became obvious. Furaha was lucky to study at secondary school because she was intelligent, and, although being female, her parents paid for her studies. However, when pregnancy was obvious, she was denied continuation by the school director. After a normal pregnancy, childbirth started spontaneously at term and she arrived at a small hospital. After a prolonged labour and signs of severe foetal asphyxia, an emergency caesarean section was made, and a large baby boy was born; however, he was already dead. Furaha had complications after, including severe pain in the pelvic region and urine passing continuously through her vagina. The harm led her to avoid social contacts, and continuing studies at secondary school was impossible. This is the situation when Furaha arrived at the Panzi Hospital eight months after childbirth.
- *Medical examination*: The gynaecologist finds a vesicovaginal fistula of 3 cm; the cervix is not visible, and the uterus is not palpable. Laboratory tests show signs of urinary infection. The gynaecologist informs and explains that the fistula can be repaired by operation.
- *Psychological examination*: Furaha meets a psychologist who listens actively to her and assesses that she is very sad and discouraged and is in need of weekly meetings with the psychologist to process her experiences further and for mental support.
- *Appointments with different professionals*: The social assistant has regular follow-ups with Furaha to verify that her file is updated and that necessary protocols are filled in and followed. She listens actively and encourages Furaha to express her feelings and discloses carefully about the needed gynaecological surgery.
- Furaha believes that the fistula is due to sexual violence but is informed that it is due to a complicated and not optimally managed childbirth. After answering questions and having enough time to consider this, Furaha decides to sign an informed consent for the suggested surgery, which is then planned to occur in 1.5 months. While waiting, Furaha stays outside the hospital at the One-Stop Centre Transit accommodation and its house for women with fistulas in the vesicovaginal rectal area. She receives treatment for urinary infection and has weekly appointments with the psychologist and also with the social assistant for follow-up of her care and activities from a holistic perspective. This includes an appointment with the juridical clinic for information and discussion around the perpetrators of the sexual violence (she decides not to pursue this) and the definition of Furaha’s socioeconomic needs. Each day she participates in different life skill activities, such as learning to knit and sew, seminars about microfinancing, and other activities such as the morning session of singing and worship.
- The surgery succeeds; the fistula is fully repaired and Furaha can urinate normally. In the post-surgery episode, she continues to meet the psychologists, participate in the socioeconomic activities, and successively regain her physical and mental health. In agreement with her parents, who also have been in contact with the One-Stop Centre social assistant, Furaha decides to go back to her village and finish the remaining years at secondary school. Her vision is to study at university. As her parents cannot afford to support her economically, she plans to be a member of one of the microfinance clubs established at her village. By investing in the One-Stop Centre’s “microfinancing activities,” she plans to save money to buy a sewing machine to make clothes as a way of financing her university studies.

### The One-Stop Centre Model of Care on Societal Level

Raising awareness and activism is a major factor in the battle against sexual violence and to facilitate the return to life in one’s village. The OSC therefore arranges activities in schools, markets, churches, and other community settings. These activities teach about human rights and promote community awareness of sexual and gender-based violence through fruitful discussions. These discussions encourage taking personal responsibility, standing against rape, and fighting to eradicate it. These community campaigns also stress the importance of transferring a victim of sexual violence to a health centre within 72 hours and that women can be treated for free at the Panzi Hospital (within the SSV project). An example of such awareness activity in society is shown in Box 2.

**Box 2. An Example of Awareness Activity in Society**

An awareness activity occurred in a village 33 km north of Bukavu because 82 girls under the age of ten were raped during a short period, and 29 of these were cared for by Panzi Hospital. According to testimony from parents and inhabitants, the perpetrators secretly entered homes at night, kidnapped the girls, took them into the bush to rape them, and abandoned them to be found later with serious genital lesions. For local community awareness and mobilisation against these child rapes, representatives of Panzi Hospital, including the medical director, and V-Men (a movement of men committed to stopping sexual violence) arranged a large delegation. Activities comprised roundtable discussions with representatives from the political and administrative authorities, police, the justice system, and the local army. Radio broadcasts also drew the attention of the governors.

The OSC organisation also trains authorised persons in society in paralegal skills for acting as informal “lawyers” for individual women and for society, working in close collaboration with heath care facility heads and community security systems (police). They report new assaults to the local security organisation and try to solve problems locally by organising meetings between offenders and victims.

## The One-Stop Centre Model Has a Right to Health Perspective

The goal of reducing inequities in health requires attention to unfair distribution of power, money, resources, and everyday life conditions [14]. This includes not only health care itself but also underlying determinants such as clean water and sanitation, adequate food, safe housing, access to education, and the possibility of supporting oneself [15]. The essential factor for health on micro (individual), meso (societal), and macro (national/global) levels is a country’s governance. This is particularly challenging in DRC. In terms of essential governance [16], DRC is among the weakest countries in terms of accountability, political stability, absence of violence/terrorism, government effectiveness, regulatory quality, rule of law, and control of corruption.

The Panzi OSC model, situated in the eastern part of Congo, is aligned with the right to health philosophy. It strengthens the position of women in society not only by treatment after their traumatic experiences but also through education and training in life skills. Furthermore, it challenges the prevailing pathological behaviour of sexual violence at the meso level at several organisational levels of society. These efforts can bring about the policy changes necessary for improving the right to health.

## Lessons Learned and the Road Ahead on Personal, National, and Global Levels

The OSC, as described here, is a holistic, person-centred care model that treats sexually, bodily, and mentally harmed women as dignified persons having major needs but also being valuable resources for their own healing and society. Their narratives are listened to, and, as persons, they are capable co-creators of their own care plan and healing. We have learned that the persons treated in this care system come out restored not only physically but also in their human dignity. Thus, the OSC model offers much more; it provides a platform for achieving a healthy life at both the micro (the person) and meso (local society) levels. It is a success that beneficiaries are integrated back into their families and communities, such as those grouped in village-savings associations or credit solidarity groups, which are patterns of entrepreneurship and mutual protection. Such groups are the future of social reconstruction in a post-conflict area.

The OSC concept, as conceived by the Panzi Hospital, was presented and adopted by the Heads of State and Government of the member states of the International Conference on the Great Lakes Region in Africa at the fourth summit and special session on Sexual and Gender Based Violence 2011 [17]. This recognition has encouraged and supported implementation of the model in some countries of the region, such as Rwanda, Burundi, and Kenya.

Despite the success of the OSC care model, challenges are not lacking, such as acceptance and integration of mental health into the primary health care system; lack of political will, which delays the replication of the OSC model; and the need for ongoing evaluations by staff on structures and protocols used, followed by revisions.

We believe that if the OSC care approach is conscientiously and systematically implemented in all health care structures, it is a strong tool to achieve the right to health for all, even in a country that lacks a health care organisation of adequate quality.

## Abbreviations

DRC: Democratic Republic of Congo
OSC: One-Stop Centre
PCC: Person-Centred Care
SSV: Survival of Sexual Violence
